# Neural network models for diagnosing recurrent aphthous ulcerations from clinical oral images

**DOI:** 10.1038/s41598-025-06951-5

**Published:** 2025-08-12

**Authors:** R. Raja Subramanian, R. Raja Sudharsan, Bavithra Vairamuthu, Deshinta Arrova Dewi

**Affiliations:** 1 Kalasalingam Academy of Research and Education, Virudhunagar, India; 2 Velammal College of Engineering and Technology, Madurai, India; 3 Faculty of Data Science and Information Technology, INTI International University, Nilai, Malaysia

**Keywords:** Deep learning, Oral ulcer, UlcerNet, Aphthous ulcerations, Fog-enabled cloud framework, Fog environment, Process innovation, Public health, Data processing, Image processing

## Abstract

In the medical field, Artificial Intelligence (AI) for diagnostic processes, particularly through deep learning techniques, has become increasingly advanced. Minor trauma, such as accidental cheek biting, sharp dental edges, or poorly fitting dentures, typically causes painful mouth ulcers and bump-like sores inside the mouth. Traditionally, diagnosing these ulcers involves a dentist or physician performing a physical examination, visually assessing the sores, and asking detailed questions about their size, location, duration, and related symptoms. Our research focuses on the advanced classification of oral ulcer stages using a convolutional neural network (CNN). To evaluate performance comprehensively, we developed and tested three custom models, comparing their effectiveness in distinguishing between different stages of oral ulcers. We also explored various optimizers and activation functions to determine the best configuration for improving model performance. Although our models show promising potential as diagnostic tools for oral ulcers, they occasionally make errors. Among the models tested, UlcerNet-2 stood out for its performance. Using the RMSprop optimizer along with Softmax and SELU activation functions, UlcerNet-2 achieved a validation accuracy of 96%. These results highlight UlcerNet-2’s exceptional effectiveness in classifying oral ulcer stages, achieving a commendable balance of high accuracy, precision, and recall. This suggests UlcerNet-2 has significant potential as an advanced diagnostic tool, possibly enhancing clinical practices in detecting and staging oral ulcers. The proposed model (UlcerNet) was implemented on FogBus, the cloud framework to empirically evaluate the model performance in cloud-fog interoperable scenarios.

**Supplementary Information:**

The online version contains supplementary material available at 10.1038/s41598-025-06951-5.

## Introduction

Mouth ulcers clinically referred to as aphthous ulcers or canker sores, manifest as painful lesions in the soft, pink mucosal lining of the mouth. These blisters cause the surrounding skin to become tense, red, and swollen, making common activities like eating, drinking, and talking particularly uncomfortable, especially when consuming spicy, salty, or sour foods^1^. Although these ulcers typically resolve spontaneously, approximately 20% of individuals experience recurrent episodes. While mirror trauma to the oral mucosa is a common etiology, other contributory factors include allergens, dental issues, certain toothpaste ingredients, bacterial infections, acidic foods, nutritional deficiencies, hormonal fluctuations, stress, and inadequate sleep. Additionally, mouth ulcers can be indicative of systemic conditions such as celiac disease, inflammatory bowel disease, diabetes, HIV, or autoimmune disorders^2,4,5^. Despite their multifactorial origins, these lesions generally undergo self-resolution. The buccal mucosa is particularly susceptible to ulcer formation, with numerous factors contributing to its pathogenesis. Secondary etiologies include specific medications, intraoral skin rashes, viral, bacterial, or fungal infections, chemical exposures, and various medical conditions. Dentists possess the expertise to diagnose and manage these oral lesions effectively. Oral ulcers exhibit a range of morphological presentations, such as reticular patterns, hollow and thin open sores, blisters, thick plaques, or nodular formations^3^. Typically, reticular, nodular, and plaque-like lesions are asymptomatic, whereas blisters, open sores, and other types of ulcers are frequently associated with pain^29,30^. Lichen planus, a specific form of oral lesion, carries a small risk of malignant transformation, with an incidence rate ranging from 0.4 to 3.7%. This condition primarily affects the mucous membranes which are found in the mouth and the skin, and treatment often follows corticosteroids by application orally or topically as treatments for symptoms and to keep the disease under control.

Catalase is a crucial enzyme for metabolic processes, but its production is reduced in catalasemia, a pathological condition [S1]. Oral ulcers, commonly known as canker sores or aphthous ulcers, are frequent and can result from minor injuries, acidic foods, or stress. Recurrent aphthous stomatitis (RAS) is a specific form of RAS, marked by painful, round or oval-shaped ulcers. Eosinophilic ulcers are benign, self-limiting conditions that typically affect the lateral side of the tongue [S2]. Detailed explanations of S1 and S2 can be found in the supplementary file.

Over 25% of the population is affected by oral ulcers, with recurrent aphthous ulcers being the most predominant. A report from India found that 92.11% of patients had minor aphthous stomatitis, 1.34% had herpetiform aphthae, and the remaining patients had significant aphthous ulcers. The causes of these ulcers may include trauma, systemic conditions, medications, or recurrent aphthous stomatitis (RAS)^6,7,28^. The prevalence of RAS was notably higher among doctors and nurses compared to individuals in other professions, with the most affected age group being 18–25 years. Individuals aged 18–24 were found to be the most susceptible to recurrent mouth ulcers. In contrast, malignant ulcers constituted 5.90% of all mouth ulcers in the 51–60 age group, while traumatic ulcers represented 4.91% in the 61–70 age group^8^.

In contrast to traditional diagnostic systems for oral lesions, contemporary methodologies leverage extensive datasets for training and testing, which can occasionally result in diagnostic inaccuracies related to the patient’s health condition. For instance, Guo et al. introduced an advanced Residual Network algorithm that exhibits promising accuracy in classifying dental ulcer images. However, its effectiveness is contingent upon the availability of thoroughly validated data^11–13^. Similarly, Fu et al. proposed and implemented a deep learning algorithm for detecting proliferative growth ulcers within the oral cavity, underscoring the critical importance of rigorous validation and training to fully exploit the model’s clinical potential. This underscores the necessity for more robust data collection and validation protocols to enhance the efficacy and reliability of deep learning algorithms in the diagnosis of oral ulcers.

Although some past research has investigated the application of deep learning to diagnose different oral conditions, such as ulcerative lesions, most are based on standard CNN architectures or domain adaptation by transfer learning from pre-trained networks like VGG-16, VGG-19, ResNet-50, ResNet-101, Inception V3, and MobileNet V2. These methods, though efficient, tend to be incomplete in adapting to domain-specific characteristics in oral ulcer images. In comparison, this research presents the UlcerNet series purpose to designed CNN architectures specifically for oral ulcer diagnosis. Through rigorous testing of various activation functions and optimizers on three variants of models, this research provides better classification accuracy with efficient models. Additionally, it compares the models proposed in this work against well-known CNNs and exhibits better or comparable performance, hence setting a new standard in the field of deep learning for oral healthcare.

Deep learning holds significant promise in the diagnosis of oral ulcers. Advanced deep learning models for classifying and recognizing various types of oral ulcers often outperform human doctors, particularly in cases where macroscopic characteristics are highly similar. By leveraging deep learning, a less invasive, cost-effective, and rapid method for early diagnosis of oral cancers, including oral cavity squamous cell carcinoma, can be achieved, facilitating timely intervention. Furthermore, deep learning-based automated tools are likely to provide valuable pre-sorting assistance to physicians, especially in urgent cases. Additionally, deep learning facilitates objective measurement of lesion severity and treatment progress, addressing the complexities inherent in subjective evaluation. In summary, the diagnostic precision, speed, and ability to quantify offered by deep learning make it an exceptional tool for evaluating oral ulcers, reducing the limitations associated with manual expert assessment^10,11,14^.

This paper presents several key contributions:

- Our proposed model, UlcerNet, achieves 96% validation accuracy in identifying oral ulcer affect in comparison with our custom models taken in the comparative study.
- In this comparison, we validated three custom models with respect to UlcerNet to find the most efficient CNN architecture for classification of oral ulcers.
- We measured its effectiveness in various key performance indicators, including accuracy, precision, recall, and finally the F1-score. We further went ahead to evaluate it with precision-recall curves, as well as confusion matrices.
- The FogBus framework has been used to deploy the UlcerNet model. Here, we have analyzed various performance metrics: arbitration time, execution time, jitter, and latency. All of them were done with a motive to predict whether a patient with RAU falls into one of five classifications: herpetiform ulceration, infectious ulcers (e.g., TB and HMFD ulcers), major RAS, minor RAS, and OSCC.

## Literature survey

This section reports on the detailed literature survey of the work that has been done in the field of oral ulcer diagnosis using deep learning techniques. We go through the review of some studies in the hope of presenting the most recent state-of-the-art methodologies that can be used to confront the pending challenges and opportunities in this domain. By synthesizing findings from various sources, we look forward to laying the foundation for our own research contribution and pointing out areas where further research is still needed.

Javed Rashid et al.^16^ pointed to the improvements in digital tools such as AI, assisted surgical techniques, machine learning, and non-invasive imaging modalities for the diagnosis and detection of oral and dental diseases, including cancer and periodontal disease (gum infection). The lips or oral cavities cancer is of a common pattern in individuals with precancerous disorders, such as PMD and OSMF. Deep learning for the latter group is a diagnostic tool that is based on the inceptionResNetv2 algorithm and has an accuracy rate of 99.51%. The research found a new database of Oral and Mouth Diseases (MOD), which consists of seven concrete data types, and adopted transfer learning to ensure consistency with the new data. In the MOD dataset, seven sub-domains were segmented, and a multitude of data augmentation schemes were used. The efforts of the future should be directed at developing experiments that would generalize more diseases in a more similar and relevant dataset.

Anushree Tiwari et al.^19^ emphasized the value of artificial intelligence (AI) in diagnosing diseases, predicting prognoses, and creating personalized treatment plans. Their study focuses on using AI to diagnose various types of mouth ulcers, following PRISMA standards. AI, applied to clinical image samples for oral ulcer classification, outperformed traditional convolutional neural network techniques in accuracy. AI was also utilized in the detection of oral cavity squamous cell carcinoma (OCSCC) through an automated, cost-effective, and painless system for evaluating cancer treatment effectiveness. Additionally, the researchers identified factors linked to recurrent aphthous ulcers (RAU) suitable for developing an AI-based predictive model. AI’s ability to improve diagnostic accuracy can lead to earlier detection, better patient outcomes, and less invasive procedures, while also enabling personalized treatment plans and streamlining workflows. AI-driven tools like chatbots and virtual assistants further enhance patient care. However, challenges such as data privacy, ethical concerns, continuous algorithm validation, and the need for ongoing education of dental professionals, alongside cost and accessibility considerations, must be addressed to fully unlock AI’s potential in dentistry.

Rita Fabiane et al.^21^ mentioned that the Artificial intelligence field has been of great help to health care, where the area of study is based on the development of convolutional neural networks for automatic classification into six different categories of clinical representation of oral lesion images. The model was developed to classify images into six elementary lesions: papulo/nodular, maculo/spots, vesicular/bullous, erosion/ulcer, and plaque. Four architectures were tested; An erroneous classification matrix served as the main metric. The model reached an average accuracy rate of 95.09% in 5069 images from different oral mucosa lesions after the process of hyper parameter optimization. The model turned good in terms of accuracy, about 71%, in all six lesion classes. As a matter of further investigation, it is imperative to identify blueprints of the trained layers so as to make a patient diagnosis easier between benign, potentially, or actually malignant tumors. The findings have already been very productive and are part of the first stage for creating a mechanism that can detect patterns in images.

Rinkal Shah et al.^22^ have believed a deep learning method using camera images as the most feasible method of detecting early-stage oral cancer, which is the most feared type of the disease and has a high mortality and morbidity rate; it’s a public health concern. It may lead to irreversible conditions and may end up leaving the patient with a permanent illness that affects both physical and mental health. The early detection of this disease is very important because the rate at which this illness spreads is very high, and only biopsy can be used as a remedy. Therefore, the early recognition of cancer cells is essential because it will stop the disease from evolving into a serious one. This research deals with obscurity in oral pathology, and it aims to solve it by using machine vision. The research demonstrates the implementation of Transfer Learning and Deep Learning in this specific issue. The research results evidenced the importance of early diagnosis without invasive methods. Thus, on the conclusion of our studies, we can now predict how different disease groups will be segregated during the diagnostic consultation of a doctor. Moreover, the model could be tweaked in order to increase the precision for oral ulcer spotting and precancerous condition detection. Though this needs a large-scale dataset from hospitals, which can be an obstacle. Such studies will open the way to the development of a powerful diagnostic system for oral cancer, which, in turn, will improve early detection of the disease before biopsy becomes a necessity.

Siddhi Lunawat Ashwini et al.^23^ proposed that a study be carried out to find out RAS frequency among students of KIMS. Out of 450 students, 350 were carriers of the RAS, with the majority of them being female. The most frequently observed site was the lower lip, with 66% of patients presenting a lower lip lesion. Stress was a cause in 56% of patients, and 81% of who had suffered from ulcers previously. The majority had duration of 6 days or less, and 80% of cases were painful. The study implies a high level of environmental and lifestyle influences involved in what was supposed to be the disease with hereditary nature. One of the key findings of the research is that the high rate of pain of 80% of these people implies that RAS is a site of discomfort. This result stresses the fact that managing pain should be an important strategy in the overall canker sore treatment program. Holistic care that covers both physical and emotional aspects of pain can promote the overall betterment of the individuals suffering from RAS.

Yu Hsu et al.^31^ addressed the worrying rise in lip and oral cavity cancer cases by employing computer vision and deep learning to improve the early detection and classification of oral mucosal lesions. Among the YOLOv7 models tested, the YOLOv7-E6 model stood out with exceptionally high precision and recall across various lesion types. The YOLOv7-D6 model was particularly effective in identifying malignant lesions, achieving impressive precision, recall, and F1 scores. Notably, incorporating coordinate attention into the YOLOv7-D6-CA model significantly enhanced the accuracy of lesion classification. This paper offers an in-depth comparison of the YOLOv7 models for classifying and triaging oral lesions, demonstrating their potential applications in both clinical settings and remote screening. However, the study also highlights certain limitations, such as the lack of dataset variability, the absence of tactile assessment data, and the need for clinical validation. Despite these challenges, the research underscores the promise of deep learning models in advancing the early detection of oral cancers.

Lucas Lacerda de Souza et al.^32^ pointed out that deep learning is being explored for differentiating head and neck high-grade lymphomas using artificial intelligence. These models analyze digitized tissue images to improve diagnostic capabilities and reduce practitioners’ workload. Current research shows high performance metrics in segmentation and detection of abnormal lymph nodes in affected patients. The primary objective is to develop models that can distinguish between various types of high-grade lymphomas, particularly in head and neck cancers. This improves diagnostic accuracy and enables timely treatment decisions. Deep learning models show promise in differentiating DLBCL and Burkitt lymphomas, enhancing diagnostic accuracy for pathologists. However, issues concerning data quality and model generalizability remain critical for widespread clinical adoption.

Shaimaa O. Zayed et al.^33^ advocated for the integration of machine learning (ML) through artificial intelligence (AI) as a powerful tool for clinicians and oral pathologists in diagnosing a broad spectrum of conditions, including potentially malignant lesions, oral cancer, periodontal diseases, salivary gland disorders, oral infections, and immune-mediated conditions. AI’s ability to detect micro-level features beyond human visual capacity addresses complex diagnostic challenges. The DODS program, in particular, is identified as a highly reliable diagnostic tool, with regular updates incorporating new oral disease images critical to improving its accuracy, sensitivity, and specificity. Additionally, DODS can significantly contribute to medical education by providing students with problem-solving scenarios that enhance their differential diagnosis skills. While AI holds transformative potential, the irreplaceable value of human expertise underscores that hybrid models, combining AI with human oversight, will offer the most effective, flexible, and reliable diagnostic support.

Specifically, the authors, Sung‑Jae Lee et al.^34^, pointed out that OPMDs increase the risk of oral cavity cancers, especially those of the tongue. Accurate early detection by screening significantly reduces cancer-related morbidity and mortality. Deep learning technology has been explored for this purpose, although getting around several challenges, such as class imbalance problems, remains to be accomplished. This paper proposes a novelty artificial intelligence model that can improve the classification performance on the imbalanced dataset of tongue lesions. In this paper, the authors employed the Efficient Net architecture coupled with mosaic data augmentation, soft labelling, and curriculum learning strategies. They obtained an accuracy rate of 0.9444, which outperformed traditional models. This work will therefore lead a basic path for future studies on handling imbalanced data in medical imaging.

Burcu Tiryaki et al.^35^ employed Deep Convolutional Neural Networks (DCNNs) to classify tongue images for lesion detection in a dataset of 623 patients. The dataset consisted of images categorized into coated tongue, geographic tongue, fissured tongue, and median rhomboid glossitis classes. The study assessed the performance of VGG19, ResNet50, ResNet101, and GoogleNet architectures using a novel fusion-based majority voting (FBMV) technique. ResNet101 achieved the highest accuracy in binary classification, while VGG19 performed best in five-class classification. To handle class imbalance in the moderately sized dataset, the researchers utilized a transfer learning approach. The FBMV method delivered over 95% accuracy in binary classification and 88% in five-class classification. Future research will focus on expanding the dataset, further addressing class imbalance, and incorporating additional lesion categories.

Surveys^25–27,38^, conducted in 2021 and 2022 on the application of deep learning models for detecting oral ulcers have been explored by various authors, including Warin et al. [S3], Yu-Hsueh Wu et al. [S4], Corinne Legeret et al. [S5], Jianbin Guo et al. [S6], and Disha Sharma et al. [S7]. Detailed explanations of S3, S4, S5, S7, and S8 are available in the supplementary file.

### Background related work

In the above section, different deep learning models were discussed, and in this proposed work, we deploy our model in fog computing, also referred to as fogging or edge computing, which is an extension of cloud computing to the edge of a network. This paradigm brings computation, storage, and networking services closer to the end devices, reduces latency, and significantly improves application performance. Fog computing is particularly well-suited for applications that generate vast amounts of data that need to be processed in near real time.

In essence, fog computing addresses many challenges:

#### Latency

The only feasible approach to reducing latency to a significant extent, due to the processes associated with transmitting data to and from the cloud, is by processing that data closer to the point of origin.

#### Bandwidth

It relieves the network bandwidth as it processes data locally.

#### Reliability

The system is more robust because of local processing due to the fact that it does not rely highly on central cloud services, which may cause outages at times.

#### Data privacy

The processing of sensitive information locally enhances privacy and security.

Overall, fog computing represents a giant leap forward in the architecture of distributed computing and would offer responsive, efficient, and scalable solutions for a wide variety of applications—from smart cities and industrial IoT to healthcare and autonomous systems. As the research and development begin, in many ways, fog computing will go on to play a lead role in shaping up the future of processing and managing data in this highly connected world.

**Table 1:** Taxonomy of oral ulcer in different locations affected in the mouth.

Work	Buccal mucosa	Tongue	Gums	Soft palate	Hard palate	Floor of mouth	Lower up	Retromolar trigone	Lips	Cheeks	Tonsil	Labial mucosa
Oral potentially malignant disorders (OPMD) ^[15]^		✓	✓						✓	✓		
Mouth and oral disease (MOD) ^[16]^		✓	✓	✓	✓				✓	✓		
Oral ulcerative lesions in COVID-19 patients ^[17]^	✓	✓	✓		✓				✓		✓	✓
Oral ulcers in children ^[18]^		✓				✓			✓	✓		
Artificial intelligence’s use of mouth ulcers ^[19]^		✓						✓	✓			✓
Identification of oral precancerous and normal mucosa ^[20]^	✓		✓		✓	✓			✓			
Classification of elementary oral lesions ^[21]^	✓	✓	✓									
Non-invasive primary screening of oral lesions ^[22]^			✓				✓			✓	✓	
Characteristics of recurrent aphthous stomatitis in a targeted demographic^[23]^	✓					✓			✓	✓		✓
Classification on oral ulcer images with residual network ^[24]^		✓		✓			✓				✓	
Proposed work	✓	✓	✓		✓	✓		✓	✓	✓	✓	✓

From the above Table 1, titled “Taxonomy of Oral Ulcers in Different Locations Affected in the Mouth,” categorizes various studies based on the locations within the mouth where oral ulcers are found. A detailed explanation of the table is provided in the supplementary file^[S8]^.

**Table 2:** Taxonomy of oral ulcer in 2024 recent paper.

Author name	Algorithm	Accuracy	Precision	Recall	F1-score
Rinkal Shah ^22^	Mobile NET	92%	96%	92%	94%
Yu Hsu ^31^	YOLOv7-E6	66%	72%	66%	69%
Lucas Lacerda de SOUZA ^32^	Xception	95%	96%	95%	95%
Shaimaa O. Zayed ^33^	Support vector machine (SVM)	82%	–	84%	–
SungJae Lee ^34^	Grad CAM-based on ROI extraction	94%	83%	78%	76%
Burcu Tiryaki ^35^	VGG19, ResNet50, ResNet101 and GoogleNet	91%	–	–	89%
Proposed Work	UlcerNet-2 model (custom)	96	97	96	96

Table 2 presents a comparative analysis of various algorithms employed for the detection and classification of oral ulcers, based on recent research from 2024. This table summarizes findings from different studies, detailing the algorithms utilized—such as MobileNet, YOLOv7-E6, Xception, SVM, and Grad-CAM, as well as combinations like VGG19, ResNet50, and GoogleNet. It includes corresponding performance metrics: accuracy, precision, recall, and F1-score. Notably, the proposed model, UlcerNet-2, achieves the highest accuracy at 96%, along with balanced precision, recall, and F1-scores, all ranging between 96% and 97%. This performance surpasses that of other models, including MobileNet and Grad-CAM, which, while demonstrating commendable results, exhibit slightly lower scores in certain metrics.

## UlcerNet model

The following section outlines the diagnostic accuracy testing conducted to evaluate the methods and efficiency of deep learning algorithms for identifying oral ulcers. It details our approach to data collection, model development, and evaluation, drawing on established methodologies and prior research. A comprehensive description of these methodologies ensures that our research results are transparent, reproducible, and conducted in a systematic and rigorous manner, rather than being arbitrary. We also discuss the modifications and improvements made to these methodologies and the rationale behind them. By employing a systematic and structured approach, we aim to make significant contributions to the field of oral ulcer diagnosis using deep learning techniques.

**Fig. 1:**
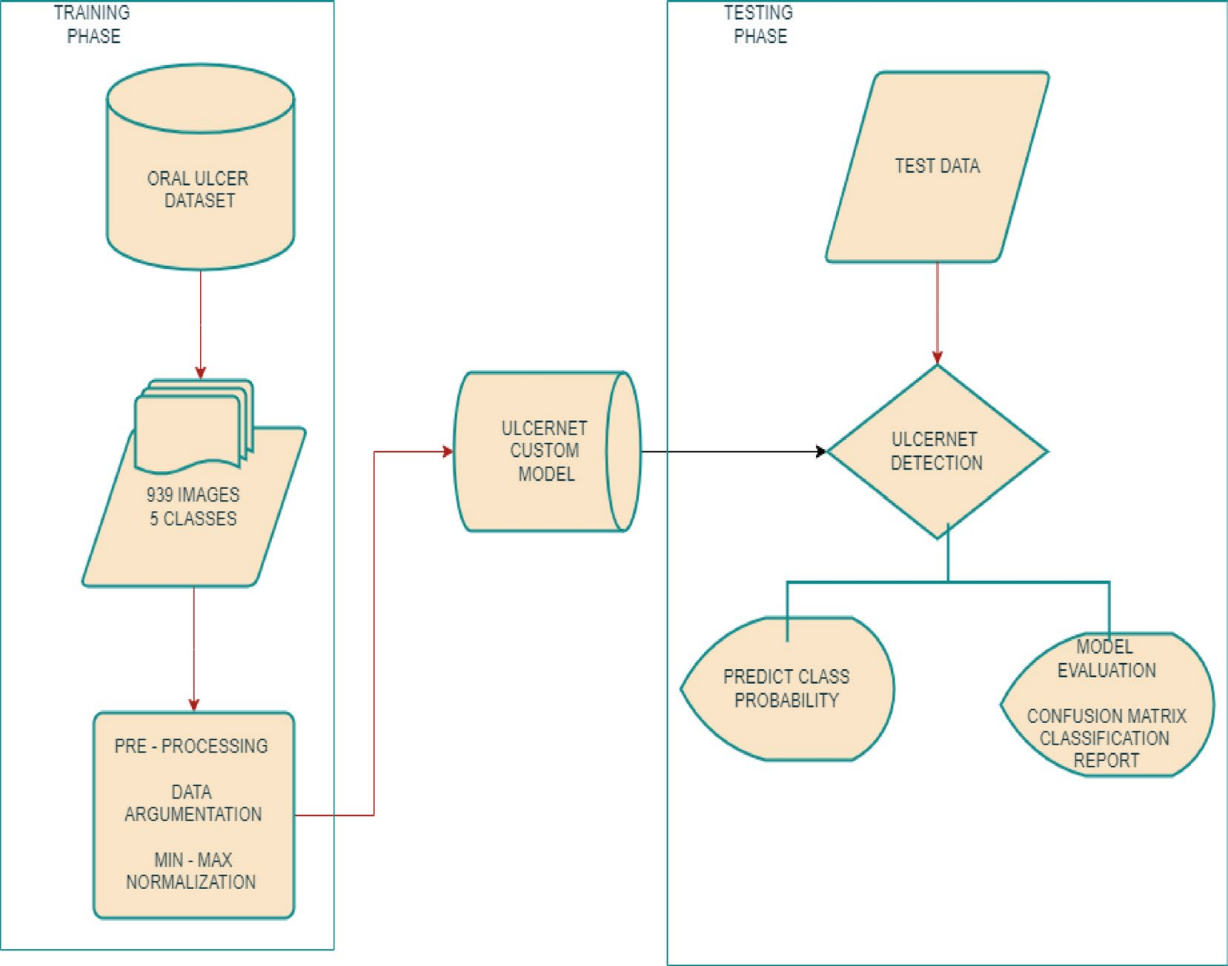
The figure below demonstrates the whole process of identifying oral ulcers.

Figure 1 illustrates the structure of the oral ulcer detection model, with all components clearly depicted. We begin with data collection, which is sourced from Roboflow, an open-source platform highly regarded for scientific studies on oral ulcers. The next step involves analysing the data, including preprocessing to format it appropriately for model use. This pre-processing encompasses normalization and resizing, preparing the dataset for subsequent stages. We then propose models tailored to our dataset, designed to accurately classify oral ulcer stages in pre-processed fundus images, thereby ensuring stable and consistent detection.

To address model calibration, we use data on unknown oral ulcers. Our oral ulcer detection system categorizes probabilities for the identified classes. We introduce the UlcerNet model, our proposed solution to overcome challenges not effectively addressed by previous models. The primary objectives of UlcerNet’s development are to enhance performance, improve categorization in oral ulcer diagnosis, and establish its applicability in the medical field as a pioneering and effective model.

### Data acquisition

In our study, we utilized the Oral Ulcer dataset^37^, which consists of 939 images sized at 224 × 224 pixels. These images are classified into five categories: herpetiform ulceration, infectious ulcers (including TB and HMFD ulcers), major recurrent aphthous stomatitis (RAS), minor RAS, and Oral Squamous Cell Carcinoma (OSCC). We allocated 80% of the images to the training set, while the remaining 20% were divided equally between the test and validation sets, each containing 94 images. The validation set is essential during the training process, as it is employed to fine-tune the parameters of our proposed model and enhance its performance, thereby mitigating the risk of overfitting. After completing the training phase, we utilize the test set to assess the model’s overall performance.

**Table 3:** Oral ulcer dataset’s description.

Name of the classes	Number of images
Herpetiform ulceration	196
Infectious ulcer Tb and HMFD ulcer	157
Major RAS	192
Minor RAS	198
OSCC	196

Table 3 states each of the classes in the dataset corresponding with their number of images in it.

### Data preprocessing

Image pre-processing is essential to prepare data for deep learning network models, enabling them to read and process the data efficiently. The primary objective is to transform the data, ensuring the image data is comprehensible within the context of the model. During this stage, we applied normalization, which adjusts the pixel values of an image to fall within the range [0, 1]. This was achieved by dividing the pixel values by 255, thus standardizing the data for optimal model performance. Additionally, Data augmentation was performed only on the training set to combat class imbalance by raising the representation for the underrepresented classes to equal 1500 images per class. The augmentation techniques used were 90°, 180°, and 270° rotations, horizontal flips, and vertical flips, equally across classes needing additional augmentation. For each original image chosen, we created five augmented versions. This approach maintained balanced representation across the categories while preserving the validation and test set that remained untouched to assess performance in an unbiased manner.

### Hyperparmeter tuning

This study conducted manual tuning of several hyperparameters, with the learning rate, batch size, and number of epochs as the main hyperparameters. Initial values were derived from previous literature and subsequently adjusted according to validation accuracy. For the learning rate (0.0001), batch size (16), and epochs (30), used empirical performance to select these values. There was no automated search (e.g., grid search, random search, or Bayesian optimization). These values were chosen based on prior experimentation and validation performance.

## Proposed work

The architectural design of the proposed UlcerNet models is based on both empirical experimentation and the inherent complexity of the oral ulcer image dataset. Each model incorporates three convolutional layers to extract spatial features at various hierarchical levels while keeping the model size manageable to train on reasonably sized medical image datasets. The increasing number of filters (32, 64, 128) between Conv2D layers was designed to extract more abstract and complex features moderately. Following standard practice, each Conv2D layer used a kernel size of 3 × 3 and a stride of 1 with ‘valid’ padding, which led to early spatial dimensionality reduction after each convolution. Each convolutional layer was followed by a Max Pooling layer with a pool size 2 × 2 to down sample the feature maps and reduce the computational load. A Dense layer with 1024 neurons was used as a fully connected layer to create sufficient capacity for possible learning of non-linear combinations of high-level features, followed by a dropout layer (rate = 0.2) to reduce the likelihood of overfitting. The Softmax output layer classifies images into five diagnostic categories.

**Fig. 2:**
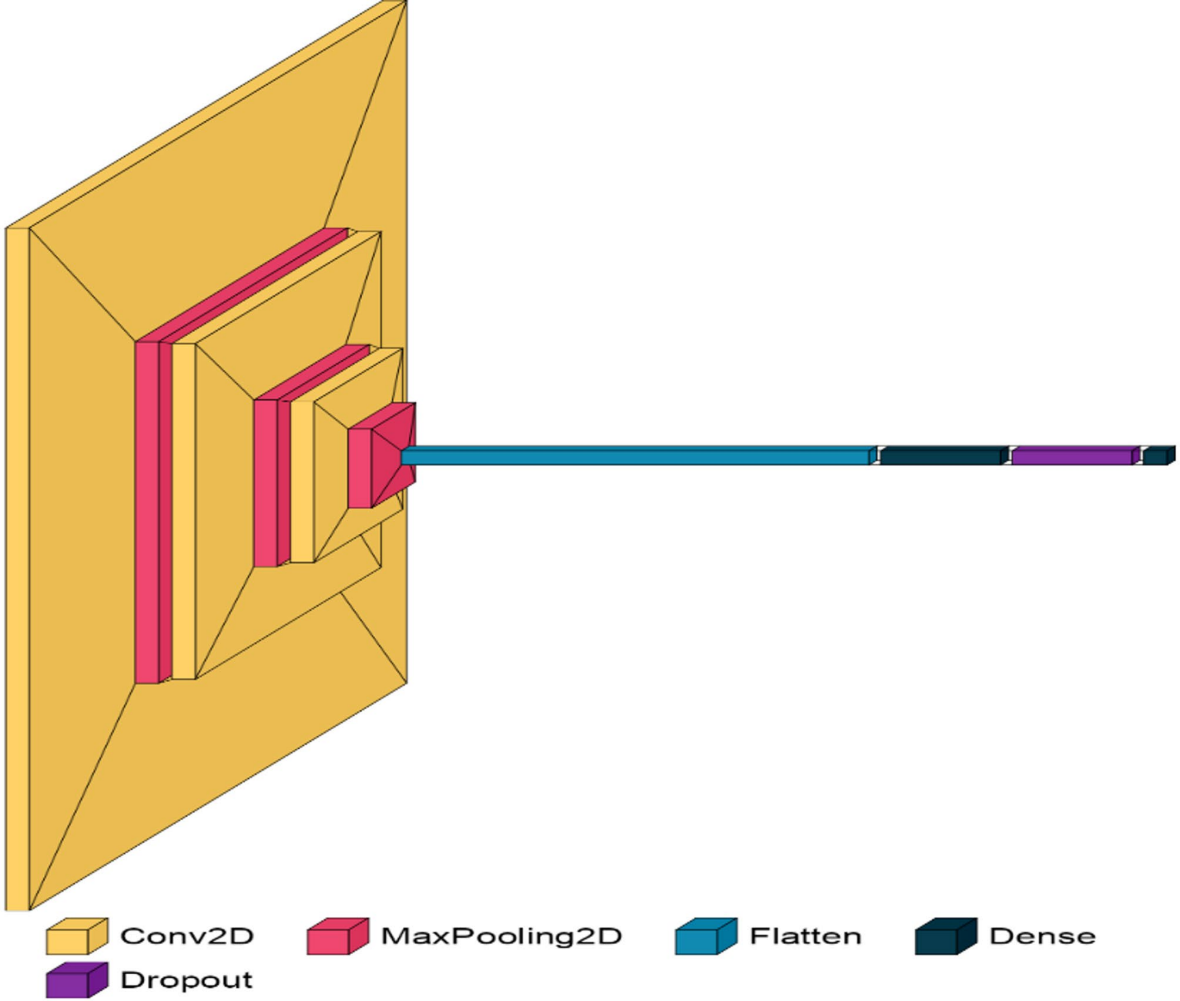
Architecture of UlcerNet-2.

The majority of oral ulcer cases affect the gums, the inner side of the cheeks, and the lips. If the symptoms are not detected in time, they can eventually lead to mouth cancer. Our suggested UlcerNet model demonstrated high quality in terms of inference and accuracy. The proposed architecture of the UlcerNet model consists of 10 stages. The key components include three Convolutional Layers, three Max Pooling Layers, two Dense Layers, one Flatten layer, and one Dropout layer. Figure 2 shows a visual representation of the proposed model.

The components of the architecture are presented as follows.

### First layer: Conv2D layer: conv2d_18

In this convolutional layer, the input image (or feature map) is processed using 32 filters, each of size 3 × 3. This operation is performed across the entire input image, creating 32 separate feature maps that highlight different patterns, such as edges or textures. The convolutional filters slide (or convolve) over the input image to compute these feature maps. The result is a set of 32 feature maps of size 242 × 242, which represent the detected features at various spatial locations. The total number of parameters in this layer includes the weights for each filter and the biases added to the output, leading to a total of 896 parameters.

### Second layer: MaxPooling2D layer: max_pooling2d_18

Following the convolutional layer, this max pooling layer performs down-sampling on the feature maps. It applies a 2 × 2 pooling window to each feature map, taking the maximum value from each window and reducing the spatial dimensions of the feature maps by half. This reduces the size of each feature map from 242 × 242 to 121 × 121 while keeping the number of features maps the same (32). The primary purpose of max pooling is to reduce the dimensionality of the data and computational complexity, as well as to provide some degree of translation invariance.

### Third layer: Conv2D layer: conv2d_19

This convolutional layer applies 64 filters of size 3 × 3 to the output from the previous max pooling layer. Each filter is used to create a new set of feature maps that capture more complex patterns in the down-sampled feature maps. The output consists of 64 feature maps, each sized 119 × 119. This layer’s parameters include the weights for each of the 64 filters and their biases. With 18,496 parameters, this layer helps the network learn more abstract representations of the input data.

### Fourth layer: MaxPooling2D layer: max_pooling2d_19

This max pooling layer follows the second convolutional layer and further reduces the spatial dimensions of the feature maps. It uses a 2 × 2 pooling window to take the maximum value in each window, reducing the size of each feature map from 119 × 119 to 59 × 59. The number of feature maps remains unchanged at 64. Max pooling helps in reducing the computational load and in abstracting the feature representations by making the output invariant to small translations and distortions.

### Fifth layer: Conv2D layer: conv2d_20

In this convolutional layer, 128 filters of size 3 × 3 are applied to the output from the previous max pooling layer. Each filter generates a new feature map, resulting in 128 feature maps of size 57 × 57. This layer is crucial for detecting even more complex features in the data. The large number of parameters (73,856) reflects the increased complexity and capacity of the network to learn detailed and abstract features from the input.

### Sixth layer: MaxPooling2D layer: max_pooling2d_20

This max pooling layer continues the process of down-sampling by applying a 2 × 2 pooling window to the 128 feature maps of size 57 × 57, resulting in feature maps of size 28 × 28. This further reduces the spatial dimensions and the number of computations needed while preserving the most prominent features detected by the previous convolutional layers.

### Seventh layer: flatten layer: flatten_6

The flatten layer converts the 3D tensor (28 × 28 × 128) into a 1D vector of length 100,352. This step is necessary for transitioning from the convolutional and pooling layers, which operate in a spatial domain, to the fully connected layers that perform classification or regression tasks. The flatten operation ensures that all features from the previous layers are used as input to the dense layers.

### Eighth layer: dense layer: dense_12

This fully connected (dense) layer consists of 1024 neurons, each connected to every element in the flattened vector. It is responsible for combining the features extracted by the convolutional and pooling layers into a more compact and abstract representation. Each neuron computes a weighted sum of all inputs and applies a non-linear activation function, enabling the network to learn complex patterns. The large number of parameters (102,761,472) in this layer highlights its role in learning and encoding complex feature interactions.

### Nineth layer: dropout layer: dropout_6

The dropout layer is a regularization technique applied to the output of the previous dense layer. During training, it randomly sets a fraction of the input units to zero, which helps prevent overfitting by ensuring that the network does not become too reliant on any specific neuron. This layer helps improve the generalization of the model by making it more robust to variations in the input data.

### Final dense layer: dense_13

The final dense layer is the output layer with 5 neurons, corresponding to the number of classes in a classification task. Each neuron produces a score that represents the likelihood of the input belonging to a particular class. The output shape of (None, 5) indicates that the layer outputs a vector of 5 probabilities for each input sample. The number of parameters (5125) includes weights and biases used to transform the 1024-dimensional vector from the previous layer into class scores.

We have proposed three custom UlcerNet models, named UlcerNet-1, UlcerNet-2, and UlcerNet-3. I will discuss these models in detail below.

## Experimental outcomes

### Environment setup

The studies were conducted using a Kaggle Notebook, which operated on an Intel^®^ Xeon^®^ CPU @ 2.00 GHz, utilizing 8 threads for efficient multitasking, and 32 GB of RAM to support robust performance. For GPU acceleration, we selected the Tesla T4 × 2 from the NVIDIA inventory, equipped with 16 GB of GPU memory. The GPU facilitated high-precision calculations essential for the intensive nature of the tests. The combination of a high-performance CPU and GPU was critical for implementing the resource-intensive processes effectively. The Kaggle Notebook was utilized to achieve our weekly objectives within the constraints of available resources. We chose Python due to its versatility in task automation, analysis, and machine learning applications. We employed a range of methods, including the OpenCV package, which is renowned for its comprehensive set of tools for addressing computer vision challenges.

### Performance analysis

The CNN architectures proposed in this study, namely UlcerNet-1, UlcerNet-2, and UlcerNet-3, were developed from scratch and are not derived from or modified versions of existing standard architectures such as VGG-16, VGG-19, ResNet-50, ResNet-101, Inception V3 and MobileNet V2. These models were specifically designed to fulfill the oral ulcer classification requirements by considering the nature and size of the dataset, further ensuring computational efficiency.

The structure of the UlcerNet-2 model is embellished in Table 4. The goal of UlcerNet-2 is to examine and evaluate different activation functions and optimizers to enhance performance in the development of oral ulcer classification tasks.

From Table 4, UlcerNet-1, which uses the Adam optimizer with Softmax, the ReLU activation function achieved a validation accuracy of 91%, a precision of 92%, a recall of 91%, and an F1-score of 91%. The ELU activation function yielded slightly lower results with a validation accuracy of 90%, precision of 91%, recall of 91%, and F1-score of 91%. The GELU activation function performed the worst for this model, with a validation accuracy of 86%, precision of 87%, recall of 86%, and F1-score of 86%. In contrast, the SELU activation function delivered the best performance with a validation accuracy of 93%, precision of 94%, recall of 93%, and F1-score of 93%.

For UlcerNet-2, using the RMSprop optimizer with Softmax, the ReLU activation function resulted in a validation accuracy of 89%, precision of 90%, recall of 89%, and F1-score of 89%. The ELU activation function significantly outperformed the others with a validation accuracy of 96%, precision of 96%, recall of 96%, and F1-score of 96%. The GELU activation function achieved a validation accuracy of 93%, precision of 93%, recall of 93%, and F1-score of 93%. The SELU activation function matched the ELU in high performance with a validation accuracy of 96%, precision of 97%, recall of 96%, and F1-score of 96%.

The validation accuracy for UlcerNet-3, which uses the Adamax optimizer with Softmax, was 94%, the precision was 95%, the recall was 95%, and the F1-score was 95%. The ELU activation function further improved these results with a validation accuracy of 95%, precision of 96%, recall of 95%, and F1-score of 95%. The GELU activation function showed slightly lower performance with a validation accuracy of 92%, precision of 93%, recall of 93%, and F1-score of 91%. Lastly, the SELU activation function maintained high performance with a validation accuracy of 94%, precision of 94%, recall of 94%, and F1-score of 94%.

**Table 4:** Assessing the efficiency of various optimizers and activation functions.

Model	Optimizer	Activation	Val_Accuracy	Precision	Recall	F1-score
UlcerNet-1	Adam with softmax	ReLU	91	92	91	91
		ELU	90	91	91	91
		GELU	86	87	86	86
		SELU	93	94	93	93
UlcerNet-2	RMSprop with softmax	ReLU	89	90	89	89
		ELU	96	96	96	96
		GELU	93	93	93	93
		SELU	96	97	96	96
UlcerNet-3	Adamax with softmax	ReLU	94	95	95	95
		ELU	95	96	95	95
		GELU	92	93	93	91
		SELU	94	94	94	94

### Performance of proposed ulcernet models and established CNN architectures on oral ulcer classification

To assess the performance of the proposed UlcerNet models, we conducted comparative studies against several established CNN architectures commonly used for medical image classification. These architectures included VGG-16, VGG-19, ResNet-50, ResNet-101, MobileNet V2, and Inception V3. All models were trained and tested under the same conditions, utilizing the same dataset split to ensure a fair comparison. From Table 5, demonstrated that the MobileNet V2 baseline model achieved a validation accuracy of 95%. In contrast, the Inception V3 baseline model reached a validation accuracy of 94%, the VGG-19 baseline model achieved 92%, and the ResNet50 baseline model recorded a validation accuracy of 88%. UlcerNet-2 achieved a validation accuracy of 96%, while UlcerNet-3 reached 95%. Both models outperformed the baseline, indicating that the custom-built architecture for oral ulcer classification was effective.

**Table 5:** Comparison of existing model versus proposed work.

Model	Accuracy	Precision	Recall	F1-score
VGG-16	90	91	90	90
VGG-19	92	92	92	92
ResNet-50	88	87	88	87
ResNet-101	87	89	87	88
Inception V3	94	93	94	93
MobileNet V2	95	94	95	93
Proposed work (UlcerNet-2)	96	97	96	96

#### Confusion matrix examination

The confusion matrix for oral ulcer classification using UlcerNet evaluates the model’s predictions against the actual classes of each image in the test dataset. It shows the number of correctly and incorrectly classified cases. The dataset comprises 939 images distributed across five oral ulcer categories: herpetiform ulceration, infectious ulcer-Tb and HMFD ulcer, major RAS, minor RAS, OSCC.

**Fig. 3:**
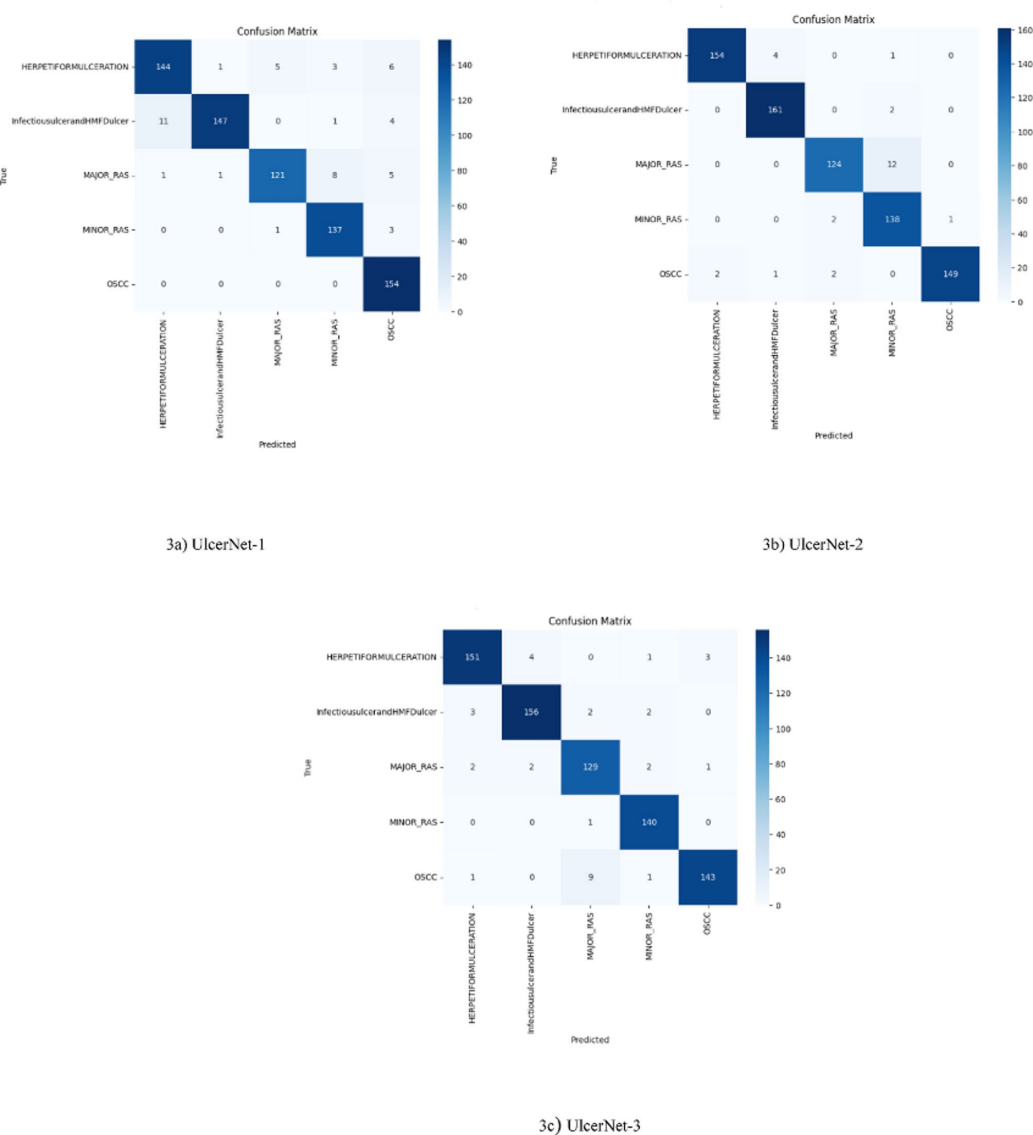
Confusion matrix of various custom model (UlcerNet).

The matrix is organized with rows representing the actual classes and columns representing the predicted classes related to oral ulcers, as shown in Fig. 3. The number of classified cases is indicated in each cell of the matrix, with deeper colors representing a higher number of images and lighter colors indicating fewer images. Diagonal cells represent correct classifications, while off-diagonal cells indicate incorrect classifications. According to Fig. 3, UlcerNet-2 (3b) predicted the true labels more accurately compared to UlcerNet-1 (3a) and UlcerNet-3 (3c).

#### Precision vs. recall examination

**Fig. 4:**
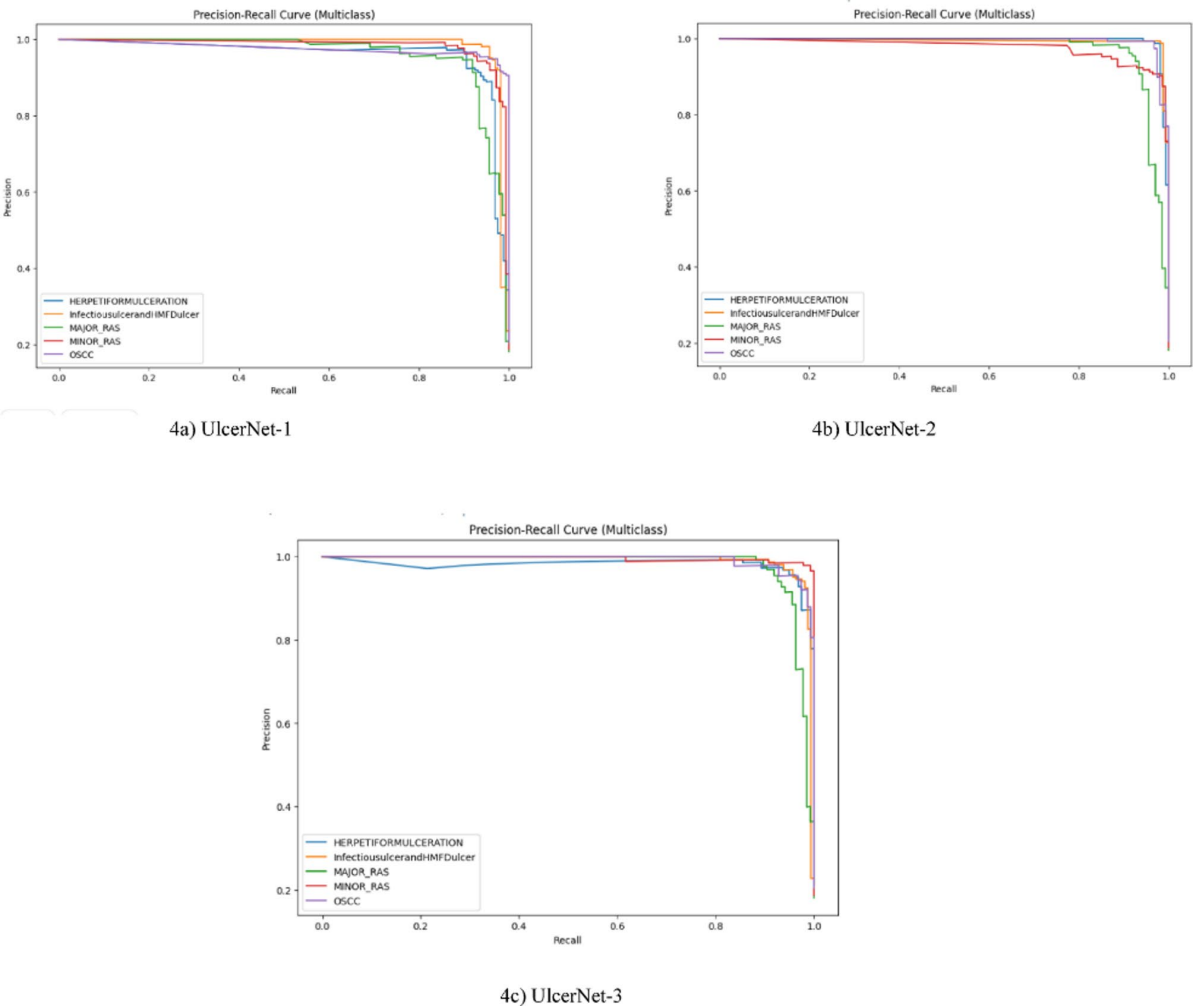
Precision vs. recall of various custom model (UlcerNet).

Precision and recall are two distinct performance metrics used to assess the effectiveness of our model. A detailed explanation, along with the equations for precision and recall, can be found in the supplementary file^[S9]^. From Fig. 4, we can see that UlcerNet-2 outperforms the other custom models (UlcerNet-1, UlcerNet-3). The x-axis of the graph denotes Recall and the y-axis denotes precision. We have also included legend, which denotes all the classes of the dataset. The main reason for calculating the precision recall curve is that we are not only depending on accuracy but also calculating precision and recall to know how efficiently our model has performed.

#### Output prediction and visual analysis

**Fig. 5:**
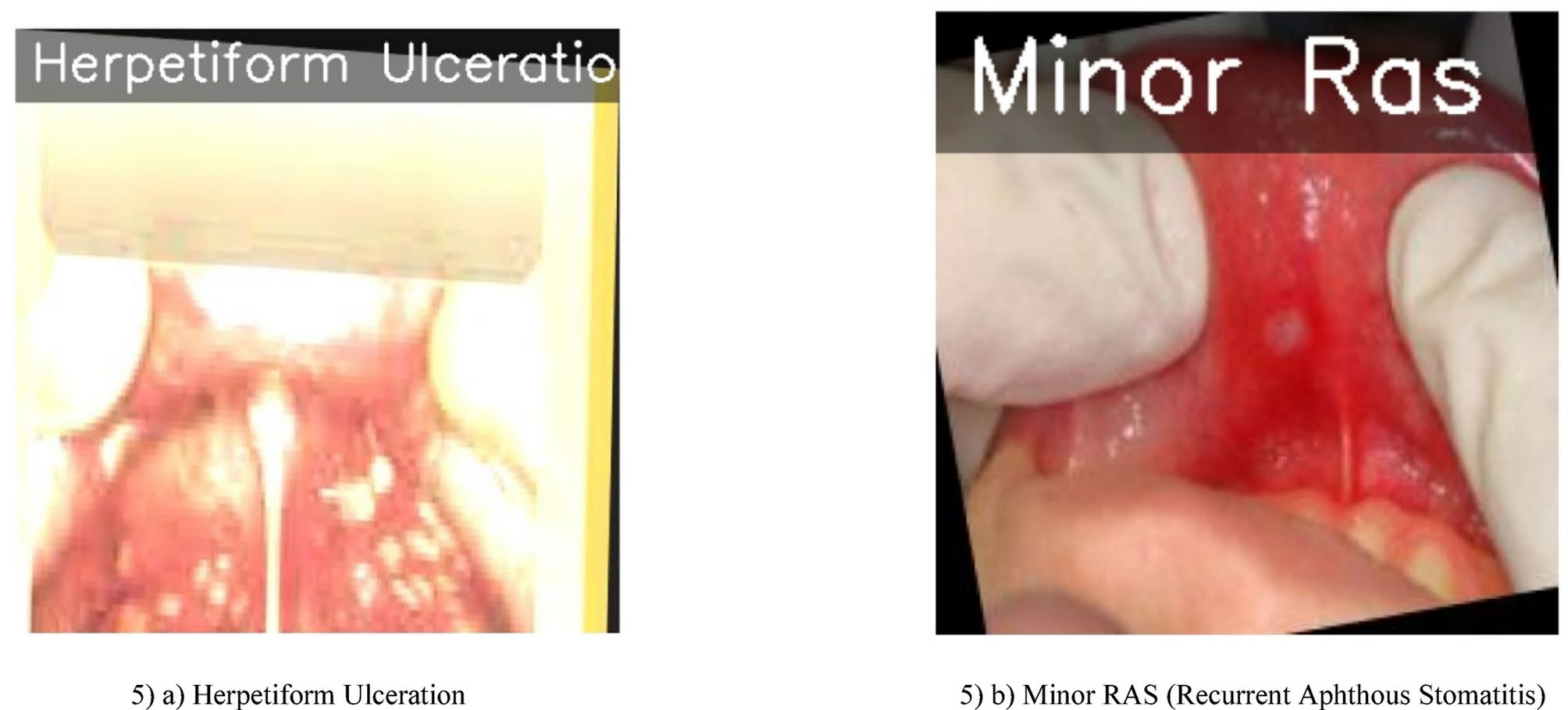
Output predictions of the proposed CNN model for oral ulcer classification.

Figure 5 displays sample predictions from the proposed CNN-based model. The images indicate how the model is capable of visually distinguishing between different types of ulcers, such as Herpetiform Ulceration and Minor Ras, thereby justifying the above classification performance metrics. These predictions demonstrate the model’s ability to confidently recognize visual patterns related to each type of ulcer. The visualization attests that the suggested CNN model generalizes well to varied ulcer presentations under different lighting and anatomical conditions, supporting its role as a diagnostic tool.

#### Outcomes of FogBus

An innovative framework called FogBus was developed to optimize the deployment of application in fog computing environments. Its unique findings include critical performance measures such as execution time, jitter, latency, and arbitration time, enabling seamless communication while proper management of available resources is facilitated. It also provides with valuable insights in terms of dependability and operational efficiency for fog-based systems.

Performance metrics such as latency, arbitration time, execution time, and jitter have been calculated using FogBus. A detailed explanation of these metrics, along with corresponding graphs, is provided in the supplementary file^[S10]^.

## Conclusion

In conclusion, this study highlights the exceptional performance of the UlcerNet-2 model in classifying and detecting RAU lesions from oral images of non-combative patients, achieving a remarkable precision of 97% and a recall of 96%. Although the UlcerNet-3 model demonstrated slightly lower performance, our results emphasize the significant promise of neural networks for automating the diagnosis of oral ulcer diseases. This technology shows potential as a valuable, non-invasive, and cost-effective tool for clinical decision-making^36^. In Table 2, we compare recent papers with our proposed work, demonstrating that our approach achieved the best results among the reviewed studies. However, it is important to note that the limited quantity of images used in this study could impact the model’s generalizability. Additionally, further empirical research is needed to validate the reliability and safety of this technology before it can be fully integrated into clinical practice. The proposed architecture can be extended and applied in a variety of other applications based on fog computing, such as agriculture, health, weather forecasting, traffic management, and smart city solutions. This can also be elaborated on the health component in order to cover the critical fields that include oral ulcer care to ensure better servicing to needy patients. These findings confirm the effectiveness of the model, thus causing a minimum of delay in the effective use of some of the complex data sets.

## Electronic supplementary material

Below is the link to the electronic supplementary material.

Supplementary Material 1

## Data Availability

The datasets used in this study include both open-access and curated datasets, with the latter being the primary focus of this research. The curated dataset can be accessed via the Roboflow Ulcer Dataset at the following link^37^, allowing users to explore and make selections as needed. Additional datasets generated or analyzed during this study are available from the corresponding author upon request.
