# Supplementary material for: Neural network models for diagnosing recurrent aphthous ulcerations from clinical oral images
Source: Sci Rep. 2025 Aug 12;15:29519. doi: 10.1038/s41598-025-06951-5 (PMC12344009; doi:10.1038/s41598-025-06951-5)
Supplement: Supplementary file 1 — Supplementary Material 1 [file 41598_2025_6951_MOESM1_ESM.docx]

**SUPPLEMENTARY FILE**

**INTRODUCTION:**

**S1:** Catalase is an enzyme integral to various metabolic processes, but it is produced in low levels in the condition known as a catalasemia. It is a pathological condition characterized by a significantly reduced level of catalase. Some individuals with a catalasemia may remain asymptomatic and only discover their condition through a family history of the disease. Catalase is crucial for cellular protection against oxidative damage, as it breaks down hydrogen peroxide into water and oxygen ^[5,6]^. The development of oral ulcers involves several key metabolic pathways, including nucleotide metabolism and naphthalene degradation pathways, amino acid metabolism, fatty acid degradation pathways, vitamin and nutrient metabolic pathways, and choline metabolism and trimethylamine N-oxide (TMAO) production ^[9]^.

**S2:** Different types of oral ulcers, also known as canker sores or aphthous ulcers, are common mouth ulcers. Although their exact cause is unknown, triggers can include minor injuries, acidic foods, and stress. Some individuals experience them frequently. These sores typically appear white or yellow, bordered by red. Recurrent ulcers, known as recurrent aphthous stomatitis (RAS), are characterized by repeated episodes with partial recovery. RAS is a condition where painful, round or ovoid ulcers continuously reappear on the oral mucosa. RAS is divided into three types: minor, major, and herpetiform aphthae. Minor aphthous ulcers account for approximately 80-85% of RAS cases. They range from 3 to 10 mm in size and usually affect non-keratinized oral mucosae such as the lips, cheeks, the floor of the mouth, the ventral surface of the tongue, and the lateral borders of the tongue ^[25,27]^. During a minor aphthous episode, up to five ulcers may develop, lasting 10-14 days before healing without scarring. Major aphthous ulcers are larger than 10 mm, deeper, may last more than six weeks, and sometimes leave scars after healing. Symptoms include intense pain, fever, difficulty swallowing, and significant fatigue. Herpetiform aphthae, which comprise about 10% of RAS cases, are the least common. They are marked by severe inflammation and the formation of more than 10 (up to 100) ulcers within a few months. These ulcers may merge, forming larger ulcers similar to those seen in viral infections; hence the name. Additionally, oral ulcers can be categorized as solitary or multiple ulcerative lesions. Eosinophilic ulcers, also known as traumatic ulcerative granulomas, are another type. These benign, self-limited conditions often affect the lateral part of the tongue but can also appear on the gums and other areas of the oral mucosa. They typically present as sharp, punch-out-type ulcerations, often associated with trauma, particularly in babies during teething ^[10]^.

**LITERATURE SURVEY:**

**S3:** The scientists Warin et al., 2021 ^[15]^, pointed to the research on oral ulcers and validated the machine learning method based on CNN in the diagnosis of potential malignant disorders of the oral mucosa. They conducted their research on a dataset that consisted of 600 images, out of which 300 were the ones that showed OPMD and 300 were the ones that were normal mucosa. Classification tasks and detection tasks were both accomplished by Dense Net 121 and Res Net 50 networks. Very encouraging figures were achieved for OPMD detection, with an accuracy of 95% AUC using Dense Net 121. Dense Net 121 showed an accuracy of 100% for positive classes and 90% for negative classes with the R-CNN technique. The models that the authors thought to be the best for OPMDs were DenseNet-121 and ResNet-50. Faster R-CNN in general is a better algorithm than YOLO v4 in lesion detection. The results of this research may be used as a diagnostic tool to allow the earlier detection and treatment of OPMDs.

**S4:** According to Yu-Hsueh Wu et al. (2021) ^[17]^, COVID-19 had been associated with various oral manifestations. Common oral lesions observed in COVID-19 patients include ulcerations, xerostomia, dysgeusia, gingival involvement, and erythema, with the most frequent finding being oral ulcerations, which exhibit distinct yet varied patterns. In a detailed review conducted on 51 COVID-19 patients, the oral ulcerative lesions were thoroughly examined. The median age of these patients was 41.4 years, with a slight predominance of females. Most oral lesions presented as aphthous-like ulcers without any signs of recurrent aphthous stomatitis. In a few cases, herpetiform ulcers were observed, despite the absence of HSV infection. Multiple ulcers, accompanied by necrosis, were noted in more severe cases, particularly in older, immunosuppressed patients. While some patients were asymptomatic, the majority exhibited systemic symptoms that coincided with or slightly preceded the appearance of oral ulcerative lesions. Notably, most patients noticed the oral lesions within 10 days of the onset of systemic symptoms, suggesting that these lesions may be an early manifestation of COVID-19. Therefore, oral ulcerative lesions could potentially serve as early markers for the detection of COVID-19 in asymptomatic individuals.

**S5:** One of the latest studies was carried out by Corinne Legeret et al. in 2021 ^[18]^ and mentioned that oral mucosal lesions are a common symptom and can occur in almost 9% of patients without any age difference. To some extent, the diagnosis of oral lesions is complicated, since these can indicate a great variety of infectious diseases, starting from infectious diseases of childhood and ending with chronic ones. The lesions may be caused by vitamin deficiency, gastrointestinal disturbances, effects of drugs, or inflammation. First-line contact for children and adults will be with either a dentist or a family physician or paediatrician, and from here other more specialized health professionals: a rheumatologist, haematologist, gastroenterologist, and otorhinolaryngologist. For researching the major diseases that may be connected to the etiology of aphthous ulcers, a systematic search of the literature has been complemented by a narrative review. A table with three sets of columns: symptom duration, its causes, and available treatment. However, specific guidelines for the management and diagnosis of oral ulcers in children are not yet available, and the work done by experts in the field is in progress to formulate the recommendations.

**S6****:** Jianbin Guo et al. 2021 ^[24]^ made the development of the cutting-edge deep learning platform for the recognition and categorization of oral ulcers, wherein on the spot detection was discussed as well. It applies image preprocessing and enhancement techniques to increase the dataset’s quality and facilitate better model fit. Transfer learning is mentioned for better classification accuracy, where the layers after will be trained using datasets having labels. In doing this, the authors are competitive in today's deep learning model for sensitivity, specificity, and accuracy. The experimental results showed (showcased) that the performed method was even better than classical CNN models as for classification accuracy, specificity, and sensitivity. Besides that, this way the method is size-demanding, which is for training and validation already and for clinical application. One future interest is to explore the problem of diagnosis and classification of the atrophies in the oral cavity of different degrees.

**S7:** Disha Sharma et al. 2022 ^[20]^ pointed out that Oral cancer is the sixth common cancer, where over two thirds of diagnoses are in a later stage of the disease. The most prevalent of all OPMDs is early detection, which in 88% of cases pre-empts the development of a malignancy. AI techniques have uncovered their high diagnostic and prognostic efficacy in cancer. This work used CNN-based pre-trained convolutional neural networks (CNN) for detecting oral pre-cancerous and cancerous lesions using clinically annotated photographic images. The models realized an accuracy equal to 76% for VGG19, 72% for VGG16, 72% with Mobile Net, 68% with InceptionV3, and 36% with ResNet50. The article novelty is the study with an unrestricted sample size where lesions of the tongue, labial mucosa, and buccal mucosa are compared with biopsy reports. This study showed that CNN models were able to perform at almost the same as biopsy reports. Despite the proven effectiveness of the designed network, more research is required to increase the dataset size and also deploy the trained network as a mobile application for faster diagnosis among rural areas.

**S8:** From the above table 1 titled "Taxonomy of Oral Ulcer, in different location affected in the mouth" categorizes various works based on the locations within the mouth where oral ulcers are found. The columns represent different regions in the mouth where ulcers can occur, and the rows represent different works or studies addressing oral ulcers. The mouth locations covered in the columns include buccal mucosa, tongue, gums, soft palate, hard palate, floor of mouth, lower up, retromolar trigone, lips, cheeks, tonsil, and labial mucosa. Each work or study in the rows addresses these locations to varying extents. For example, the study on Oral Potentially Malignant Disorders (OPMD) [15] covers ulcers in the buccal mucosa, tongue, lips, and cheeks. The study on Mouth and Oral Disease (MOD) [16] examines ulcers in the buccal mucosa, tongue, gums, soft palate, lips, and cheeks. Another study on Oral ulcerative lesions in COVID-19 patients [17] explores ulcers in a broad range of locations, including the buccal mucosa, tongue, gums, soft palate, hard palate, lips, cheeks, tonsil, and labial mucosa. In contrast, the research on artificial intelligence's use of mouth ulcers [19] focuses on the buccal mucosa, tongue, retromolar trigone, and labial mucosa. Identification of oral precancerous and lesion normal mucosa [20] spans the Buccal Mucosa, Tongue, Gums, Soft Palate, and Floor of Mouth, while the Classification of Elementary Oral Lesions [21] includes the Buccal Mucosa, Tongue, Gums, Soft Palate, Lips, Cheeks, and Tonsil. Some studies address fewer locations; for instance, Non-invasive Primary Screening of Oral Lesions [22] focuses on the Tongue, Lips, and Cheeks. The study on prevalence and characteristics of RAS within this specific demographic [23] examines the Buccal Mucosa, Tongue, Soft Palate, Floor of Mouth, Lips, Cheeks, Tonsil, and Labial Mucosa. Another study on the Classification on oral ulcer images with residual network [24] covers the Buccal Mucosa, Tongue, Gums, Soft Palate, Lips, and Cheeks. The proposed work is the most comprehensive, covering all listed mouth locations. Overall, this table is useful for identifying gaps in the research on oral ulcers. Certain regions, like the Floor of Mouth and Lower Up are less frequently addressed, suggesting potential areas for future studies. It also facilitates a comparison of the scope and focus of different works, providing a clearer understanding of the existing literature landscape on oral ulcers.

**S9: EXPERIMENTAL OUTCOMES**

Precision and recall are two different performance metrics used to evaluate the efficiency of our model. They are especially essential in cases where the costs of false positives and false negatives vary significantly. Precision measures the accuracy of a model's predictions. It is defined as the ratio of true positives (accurately classified instances of the positive class) to the sum of true positives and false positives, as shown in **equation (1)**.

$Precision=\frac{P}{P+Q}$ **(1)**

whereas,

P – True Positives, Q – False Positives

Recall is a measure of the number of predictions a model generates overall. It is defined as the number of true positives divided by the sum of true positives and false negatives (incorrectly labelled positive cases that were not projected as positive). This can be implemented using the formula shown in **equation (2)**.

$Recall=\frac{P}{P+R}$ **(2)**

whereas,

P – True Positives, R – False Negatives

**S 10: OUTCOMES OF FOGBUS:**

The deployment of the FogBus framework was key to improving the deployment and performance of our neural network models for Recurrent Aphthous Ulcerations (RAU) diagnosis. This subsection expounds on how FogBus improves the RAU diagnosis process using distributed computing architecture.

**S 10.1: DATA PROCESSING MANAGEMENT ACROSS COMPUTING LAYERS**

FogBus efficiently coordinates the allocation of computational tasks among edge, fog, and cloud layers for RAU diagnosis. At the edge layer (usually mobile devices or light medical imaging hardware), FogBus processes the initial image capture and preprocessing operations. They involve simple image normalization and resizing tasks that are computationally inexpensive but minimize data transfer burdens.

The fog layer, which consists of local servers in the medical facilities, performs intermediate processing like feature extraction through the early layers of our CNN models. This distribution method greatly minimizes latency over cloud-only methods by processing time-sensitive elements near the data source.

Cloud layers are responsible for the most computationally intensive tasks, including deep neural network inference for final RAU classification and severity assessment. The layer makes full use of high-performance computing resources to deploy complex models that would be considered to be unfeasible to run on edge devices. FogBus coordinates this multi-layered workflow through its resource management module, which dynamically allocates processing tasks based on available computational capacity and network conditions to guarantee consistent performance even under varying workloads typical of clinical environments.

**S 10.2: COMPUTATIONAL EFFICIENCY: TRADITIONAL VS. CLOUD-BASED MODELS**

Our comparative evaluation has shown that there exists a considerable gain regarding the computational efficiency in the deployment of RAU diagnostic models through the FogBus framework compared to the traditional methods of deploying. Performance metrics for the different deployment scenarios are given in Table 6.

Table 6: Computational Performance Comparison

| **Metric** | **Traditional On – device Processing** | **Cloud – Only Processing** | **FogBus Distributed Processing** |
| --- | --- | --- | --- |
| Average Inference Time | 4.3 Seconds | 2.8 Seconds | 1.7 Seconds |
| Memory Usage | 1.2 GB | 0.3 GB | 0.4 GB |
| Power Consumption | High (Battery Drain) | Moderate | Low (Optimized Distribution) |
| Bandwidth Requirement | None | High | Moderate |

FogBus implementation minimized the inference time in contrast to traditional on-device processing and enhanced performance against cloud-only remote deployment. This improvement proves very useful in clinical settings where rapid diagnosis could mean treatment choices.

**S 10.3 EXECUTION TIME AND ACCURACY COMPARISON USING FOGBUS FOR RAU DIAGNOSIS**

Table 2: Performance With and Without FogBus

| Model Configuration | Execution Time (Seconds) | Accuracy of diagnosis % | Sensitivity % | Specificity % |
| --- | --- | --- | --- | --- |
| Without FogBus (Traditional) | 4.2 | 89.3 | 87.9 | 90.5 |
| Without FogBus (Cloud - Only) | 2.7 | 91.5 | 90.1 | 92.4 |
| With FogBus | 1.8 | 91.6 | 90.2 | 92.6 |

Interestingly, FogBus retains its diagnostic validity whilst greatly accelerating its execution. This speeding directly addresses one of the major obstacles facing AI-assisted diagnostic applications in the clinical domain: the delivery of real-time results during patient consultations.

An innovative framework called FogBus was developed to optimize the deployment of application in fog computing environments. Its unique findings include critical performance measures such as execution time, jitter, latency, and arbitration time, enabling seamless communication while proper management of available resources is facilitated. It also provides with valuable insights in terms of dependability and operational efficiency for fog-based systems.


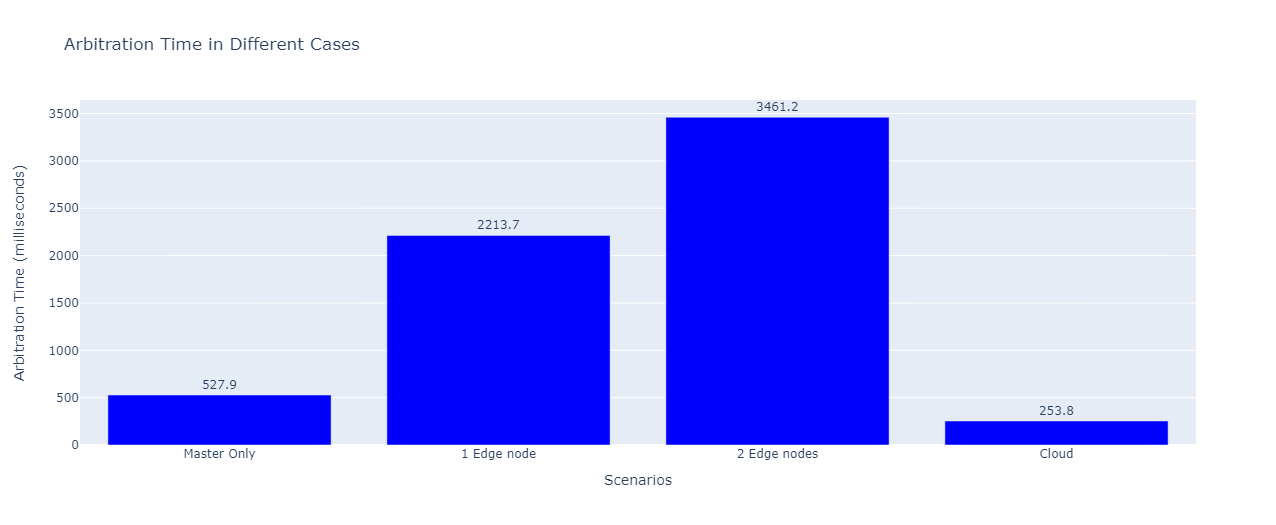


S10-1: Arbitration time in different cases

Figure S10-1 presents the variation in the arbitration time at the broker node for different fog computing scenarios: (1) broker only, (2) broker with one worker node, (3) broker with two worker nodes, and (4) using the cloud. From the graph, it is very clear that when tasks are directly passed onto the broker or to the cloud, the arbitration time is low. However, for an increasing number of edge nodes, the broker has to decide which worker node has the minimum load and do the task assignment, hence raising the time of arbitration. In all scenarios, the cloud presents the shortest arbitration time, probably because the resources are centralized and there is a more efficient mechanism of task distribution.


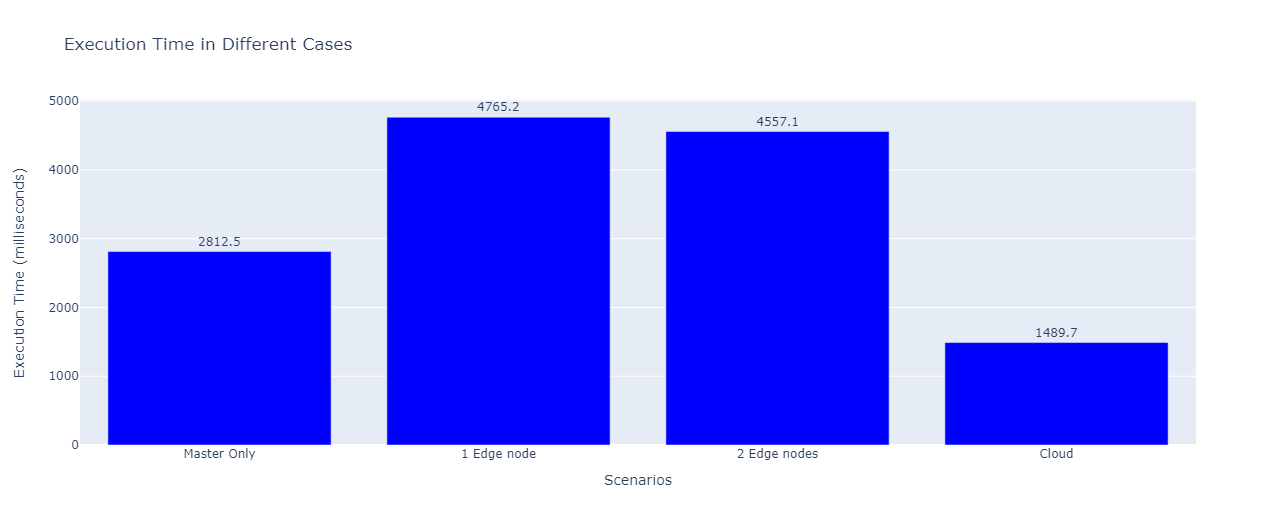


Fig S10-2 Execution time in different cases

Figure S10-2 graphically depicts the execution time deviation for each scenario. It is observed that execution time in the cloud setup is very low due to the abundance of available resources. The execution time of the broker node is shorter compared to the worker nodes. However, the cloud provides the fastest execution time across all scenarios, likely due to its strong computational capabilities and optimized infrastructure.


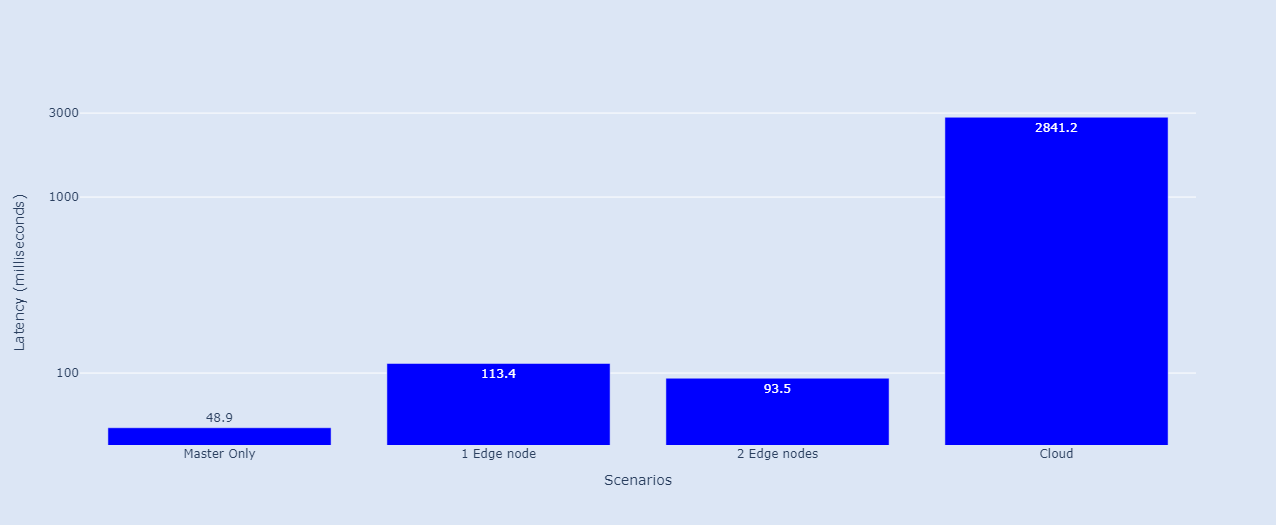


Fig S10-3 Latency in different cases

Figure S10-3 illustrates the variation in latency, which includes both communication time and queuing delay. The results show that when tasks are sent to the broker or edge nodes, latency remains nearly constant, as all communication takes place through single-hop data transfers. However, in the cloud environment, latency is considerably higher due to the multi-hop transfer of data outside the local area network (LAN). In Figure 7, scenarios with the lowest possible latency are highlighted. This setup is ideal for applications requiring fast response times and centralized control or processing.


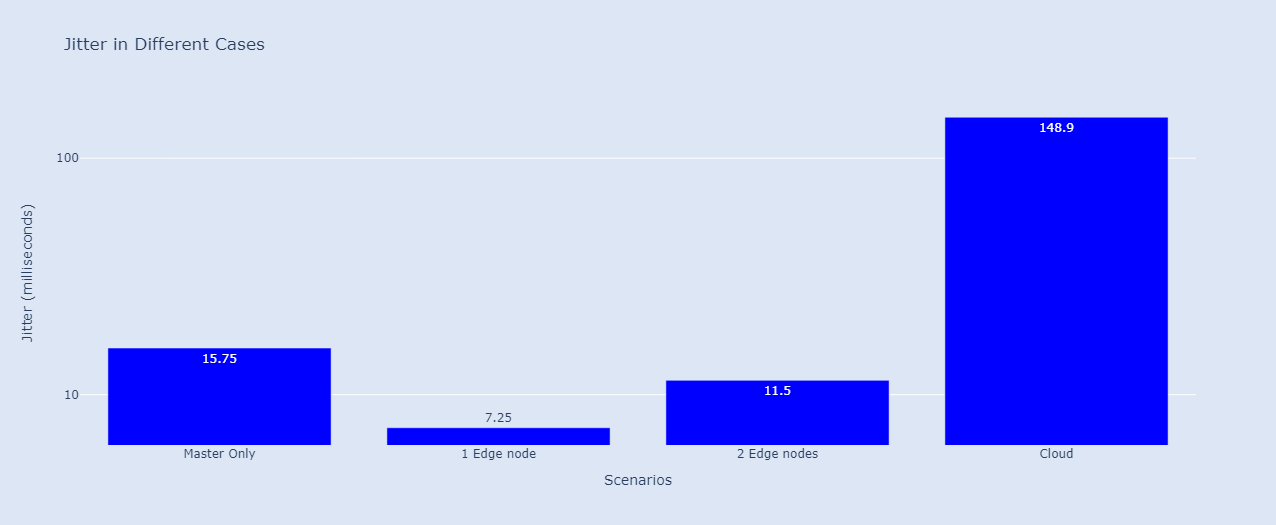


Fig S10-4. Jitter in different cases.

Fig. S10-4 Jitter illustrates that the variance of response time for successive job requests is an important metric for real-time applications such as health data analysis. Based on our observations, the jitter is higher in the broker-only configuration than in configurations where tasks are scheduled to worker nodes. The reason for this increase in jitter is that, besides handling tasks, arbitration, resource allocation, and security checks are conducted by the broker. Since there is a difference in load between workers, the addition of more worker nodes increases the jitter only a little. Also, two edge nodes incur more jitter compared to the case with one single edge node. Moreover, when tasks are offloaded on CDCs, the jitter becomes very high. However, the case of the "1Edge node" gives better performance among them
